# The Brief Kinesthesia test is feasible and sensitive: a study in stroke

**DOI:** 10.1590/bjpt-rbf.2014.0132

**Published:** 2016-01-19

**Authors:** Alexandra Borstad, Deborah S. Nichols-Larsen

**Affiliations:** 1 Division of Physical Therapy, The Ohio State University, Columbus, OH, USA; 2 School of Health and Rehabilitation Sciences, The Ohio State University, Columbus, OH, USA

**Keywords:** hemiparesis, upper extremity, somatosensation, proprioception, position sense, measurement

## Abstract

**BACKGROUND:**

Clinicians lack a quantitative measure of kinesthetic sense, an important contributor to sensorimotor control of the hand and arm.

**OBJECTIVES:**

The objective here was to determine the feasibility of administering the Brief Kinesthesia Test (BKT) and begin to validate it by 1) reporting BKT scores from persons with chronic stroke and a healthy comparison group and 2) examining the relationship between the BKT scores and other valid sensory and motor measures.

**METHOD:**

Adults with stroke and mild to moderate hemiparesis (N=12) and an age-, gender-, and handedness-matched healthy comparison group (N=12) completed the BKT by reproducing three targeted reaching movements per hand with vision occluded.

**OTHER MEASURES:**

the Hand Active Sensation Test (HASTe), Touch-Test^(tm)^ monofilament aesthesiometer, 6-item Wolf Motor Function Test (Wolf), the Motor Activity Log (MAL), and the Box and Blocks Test (BBT). A paired t-test compared BKT scores between groups. Pearson product-moment correlation coefficients assessed the relationship between BKT scores and other measures.

**RESULTS:**

Post-stroke participants performed more poorly on the BKT than comparison participants with their contralesional and ipsilesional upper extremity. The mean difference for the contralesional upper extremity was 3.7 cm (SE=1.1, t=3.34; p<0.008). The BKT score for the contralesional limb was strongly correlated with the MAL-how much (r=0.84, p=0.001), the MAL-how well (r=0.76, p=0.007), Wolf (r=0.69, p=0.02), and the BBT (r=0.77, p=0.006).

**CONCLUSIONS:**

The BKT was feasible to administer and sensitive to differences in reaching accuracy between persons with stroke and a comparison group. With further refinement, The BKT may become a valuable clinical measure of post-stroke kinesthetic impairment.

## Introduction

Stroke is a common problem worldwide^1^. More elusive than motor impairment, somatosensory impairment, in at least one modality, affects 67% of individuals with stroke^2^. Post-stroke somatosensory impairment is associated with decreased coordination in reaching and grasping^3^, decreased functional mobility, and longer hospital stays^4^. Kinesthesia is the somatosensory modality that includes limb position sense and perception of movement and is a component of proprioception. Recent research using robotics suggests that 61% of individuals with acute stroke have kinesthetic impairment^5^. It is commonly accepted that somatosensation is an important contributor to sensorimotor control mechanisms and recovery from stroke^6^; however, clinicians lack a brief and inexpensive method to quantify the active, behavioral aspect of proprioception - kinesthesia. It follows that practical, quantitative tools are needed to enable clinicians to individualize stroke rehabilitation.

The Brief Kinesthesia Test (BKT) is freely available and takes approximately eight minutes to administer^7^. For this test, error in targeted reaching tasks is measured to evaluate kinesthetic impairment. The BKT was recently shown to be valid and reliable (ICC=0.71) in healthy individuals across the lifespan^8^. The objective of this study was to determine the feasibility of administering the BKT and begin to validate it in persons with mild to moderate post-stroke hemiparesis. Here we report BKT scores from a heterogeneous group of persons with chronic stroke and a healthy comparison group and examine the relationship between the BKT scores and other valid sensory and motor measures.

## Method

### Design and participants

Using a cross-sectional design, we studied a heterogeneous sample of community-dwelling adults with chronic stroke (N=12) who were recruited through word of mouth and through advertisement with local stroke support groups. We also studied an age- (SD= 3 years), gender-, and handedness-matched healthy comparison group (N=12). Recruitment for the study was done via ResearchMatch, a national health volunteer registry that was created by several academic institutions and supported by the U.S. National Institutes of Health as part of the Clinical Translational Science Award (CTSA) program (Table 1). Inclusion criteria were a single diagnosed stroke greater than three months prior to enrollment; mild to moderate hemiparesis defined as greater than 10 degrees active extension in the contralesional fingers and wrist (required for manipulation of test objects); 45 degrees active elbow and shoulder flexion^9^; and communication in English. Potential participants were excluded if they scored less than 24 on the Mini-Mental State examination (MMSE), indicating potential difficulty following instructions; demonstrated severe spatial neglect on Albert's test^10^; demonstrated apraxia as determined by object naming on the MMSE; or reported another neurologic or sensory disorder such as Parkinson's disease or peripheral neuropathy. One control participant was excluded who reported a history of peripheral neuropathy. Participants provided written informed consent prior to participation. The Biomedical Institutional Review Board of The Ohio State University, Columbus, OH, USA, approved this study (approval number 2011H0029).

**Table 1:** - Demographic and clinical characteristics of participants.

ID	Age	Sex	Dom.Hand	Most Affected Hand	Chronicity (Months)	Lesion vascular distribution/location	BKT (cm) contralesional/ ipsilesional	Wolf (Rate metric/60 sec.)	BBT	MAL HM/HW
011	64	M	R	R	20	Lacunar/ thalamus	10.8/11	19.8	4	0.73/0.85
012	62	M	R	L	16	Lacunar/ PLIC	5.2/7.4	35.6	29	3.16/3.76
013	39	F	L	R	4	Lacunar/ thalamus	12.4/3.2	18.34	27	1.85/3.08
014	61	F	R	L	24	Lacunar/ thalamus	10.8/5.5	33.91	29	2.5/2.8
015	77	F	R	R	8	MCA/ frontal	5.8/3.4	41.88	40	4.83/4.42
016	70	M	R	L	21	Lacunar/ thalamus	4.9/7.1	53.9	52	5/5
017	60	F	R	L	94	MCA/ parietal	8/7.3	23.01	36	2.62/2.5
018	85	F	R	L	38	Lacunar/ PLIC	6/6.6	42.81	46	4.53/4.9
019	65	M	R	L	41	Lacunar/ PLIC	10.6/7.3	24.27	19	1.61/1.37
020	71	F	R	L	8	MCA/ frontal	6.6/6.9	38.5	39	3.71/3.96
021	69	M	R	R	9	MCA-ACA/ frontal	NA/5.9	1.97	0	1.83/1.33
022	48	F	R	R	13	MCA/ corona radiata	4.4/5.7	27.08	52	3.78/3.93
Mean	64	7F/ 5M	11R/1L	7L/5R	25	NA	7.8/6.4	30.09	31	3.01/3.16
Comparison (n=12)	64	7F/ 5M	11R/1L	NA	NA	NA	4.2/4.9*	NA	NA	NA

R: Right; L: Left; NA: Not Applicable; MCA: Middle Cerebral Artery; PLIC: Posterior limb of the internal capsule; ACA: Anterior cerebral artery; BKT: Brief Kinesthesia Test; Wolf: Wolf Motor Function Test; BBT: Box and Blocks Test; MAL: Motor Activity Log; HM: How much scale; HW: How well scale. *scores from the matched upper extremities of the healthy control participants.

### The Brief Kinesthesia Test and other measures

**Figure 1:**
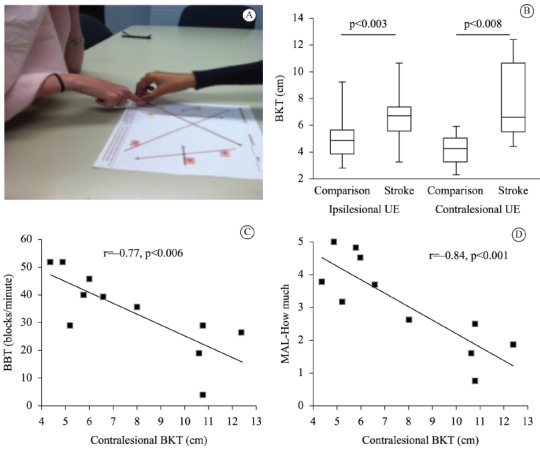
- A: BKT set-up example. B: Box plot of BKT scores (sum of the error in centimeters for the two longest reaches) for ipsilesional and contralesional upper extremity (UE). C: Pearson correlation coefficient of 0.77 (p=0.006) indicates a strong relationship between the Box and Blocks Test (BBT) and the BKT. D: Pearson correlation coefficient of 0.84 (p=0.001) indicates a strong relationship between the Motor Activity Log (MAL)-How much scale and the BKT.

The BKT was administered with the participants seated in a standard height chair (19 inches) in front of a standard height table (29 inches) with vision occluded by a curtain. Participants reproduced targeted reaching movements from a starting location to a target location on a test page after being guided by the examiner (Figure 1A). The distance from the response location to the target location was recorded in centimeters. Additional equipment needed to administer the BKT includes a visual shield (Figure 1A) and a tape measure. There were three trials per hand (two longer reaches and one shorter reach) as can be seen on the test page in Figure 1A. The BKT took an average of 8 minutes to administer including set-up. Our feasibility outcome for the BKT was whether or not participants, who met our criteria, could complete the test with standard administration^7^. Our criteria for feasibility was 90% of participants completing the test. The BKT score used here was the sum of the distance from the target in centimeters for the two longest reaches for each hand, as originally published by Dunn et al.^8^ (Figure 1B).

Other measures collected and the construct they measure are listed here. The Hand Active Sensation Test (HASTe), a measure of haptic performance of the hand, includes trials of weight and texture discrimination^11^. The Touch-Test^(tm)^ monofilament aesthesiometer is a measure of sensitivity to touch^12^, and the 6-item Wolf Motor Function Test (Wolf)^13^ quantifies upper extremity motor ability through timed and functional tasks. Wolf scores are reported using the rate metric proposed by Hodics et al.^14^, that is, the number of times on average a participant could perform the tasks in one minute. The Motor Activity Log (MAL) assesses daily use of the affected upper extremity and is based on subjective rating of how much and how well the extremity performs common tasks^15^. The Box and Blocks Test (BBT) is a test of manual dexterity in which individuals transfer 1-inch blocks from one side of a partitioned box to another. The BBT score is the number of blocks transferred in one minute^16^. Bilateral upper extremities of participants were tested on a single occasion by one examiner. For consistency, the following order of testing was used for all participants: Touch-Test^(tm)^, BKT, HASTe, BBT, Wolf, and MAL. When both hands were tested, the less affected or dominant hand were tested first.

### Statistics

A paired t-test was conducted to compare BKT scores between post-stroke and comparison groups. The alpha was set at p≤0.05. Pearson product-moment correlation coefficients were computed to assess the relationship between BKT scores and sensory and motor measures. We used the Portney and Watkins criteria for interpretation^17^. The relative standard error of the mean (RSEM) was calculated to describe the variability of the BKT scores in our sample. The magnitude of difference between post-stroke and comparison groups for BKT scores for both upper extremities was calculated using effect size. All data were analyzed using JMP^(r)^ Pro 11.0.0.

## Results

Demographic and clinical characteristics are given in Table 1. One post-stroke participant out of 12 was unable to complete the BKT with their contralesional upper extremity (UE); therefore, the feasibility criterion of 90% was met. BKT scores for both stroke and comparison groups were normally distributed. Regarding variability in BKT scores, the RSEM for the post-stroke group contralesional and ipsilesional and for the comparison group contralesional and ipsilesional were 11%, 9%, 8%, and 10%, respectively. Contralesional UE scores for the post-stroke group (n=11, *x̅*=7.8 centimeters (cm), SD=2.9, 95% CI=5.8-9.7 cm) and the comparison group (n=12, *x̅*=4.2 cm, SD=1.2, 95% CI=3.4-4.9 m) were statistically different. The mean difference between groups was 3.7 cm (SE=1.1, t=3.34; p<0.008). Cohen's effect size value (d=0.96) suggests a large practical difference between groups. Ipsilesional UE scores for the post-stroke group (n=12, *x̅*=6.4 cm, SD=2.0, 95% CI=5.2-7.6 cm) and the comparison group (n=12, *x̅*=4.9 cm, SD=1.7, 95% CI=3.8-6.0 cm) were also statistically different. Here the mean between group difference was 1.5 cm (SE=0.6, t=2.49; p=0.03). Cohen's effect size value (d=.47) suggests a low to moderate practical difference between groups. Contralateral UE BKT scores strongly correlated with the MAL-how much (r=0.84, p=0.001), the MAL-how well (r=0.76, p=0.007), Wolf (r=0.69, p=0.02), and the BBT (r=-0.77, p=0.006) but did not correlate with HASTe (r=0.355, p=0.29) or Touch-Test (r=0.095, p=0.77). In a previous study, the 95% confidence interval for BKT scores in healthy participants in the age range of our participants was 5.0 - 5.87 cm^8^. Given this, 58% of our post-stroke participants were outside of this range with their contralesional UE, while 50% were outside of this range with their ipsilesional UE.

## Discussion

Data from this preliminary study suggest the BKT is feasible to administer and may be a useful tool to identify kinesthetic impairment in individuals with mild to moderate post-stroke hemiparesis. To our knowledge, this is the first report of a clinically practical tool with the potential to quantify UE kinesthesia post-stroke. Kinesthetic awareness informs movement; therefore, it is not surprising that a strong relationship was identified between the BKT and participants' subjective rating of how well their arm performs tasks (MAL) and the objective measures (Wolf and BBT). It is also possible that a participant's ability to generate motor output affected the BKT scores in this study. Determining the discrete somatosensory and motor contributions to this sensorimotor task is an area for future research.

Post-stroke somatosensory impairment is difficult to recognize unless the loss is profound. The nature and extent of post-stroke somatosensory impairment has been difficult to describe due to a variety of factors including: 1) stroke heterogeneity; 2) multiple somatosensory modalities; 3) multiple body areas effected; and 4) scarce quantitative measurement tools. Our finding that BKT was not related to touch perception or haptic performance is consistent with evidence that there is low agreement between modalities in post-stroke somatosensory impairment^18^. Touch perception threshold is low in the somatosensory hierarchy^19^ and reflects cutaneous receptor function of the index finger without a movement requirement. The HASTe is a measure of haptic performance, which requires cutaneous receptors of the hand, proprioceptive receptors of the UE, and active movement and is an example of a higher-level somatosensory process^19^. The BKT would also be considered higher level, given that scaling of distances is required with proprioception of the UE and active movement is essential to the task. Therefore, while the BKT shares with the HASTe the requirement of UE proprioceptive information, it differs from both other somatosensory measures used here in that cutaneous input will not drive performance. As with all studies of this size, interpretation should be done with caution.

In comparison to the commonly used method of joint position matching^20^, the BKT has the following advantages. First, the BKT has a ratio scale of measurement. BKT scores from this study were normally distributed and ranged from 3.2 to 12.4 cm error for post-stroke and from 2.3-9.2 cm error for comparison participants. Ratio scales of measurement are unambiguous and allow all mathematical and statistical operations^17^. Second, normative data are available by age group^8^, eliminating the need to compare to the ipsilesional extremity, which may be problematic as discussed below. In comparison to robotics^5^, the primary advantage of the BKT is that it is freely available and does not require special equipment to administer, therefore being practical for therapists in settings worldwide. The primary disadvantage is that the BKT scores are based on localization of the target; other metrics that may reflect kinesthesia, such as velocity of movement and smoothness of reaching trajectory and response latency, are not quantified with the BKT. Comparisons in larger studies using a gold-standard measure of kinesthetic impairment, such as robotics^5^, are needed to further validate the BKT as a method of quantifying post-stroke kinesthesia.

Importantly, the exact contribution of motor and somatosensory impairment to BKT scores in this study is uncertain. This is the primary limitation of attempting to quantify kinesthetic impairment using a targeted reaching task in this population. This limitation applies to simple tests, such as the BKT, as well as to sophisticated approaches such as robotics^5^. After stroke, poor reaching accuracy may be due to limited motor output versus poor control of movement secondary to impaired kinesthetic sense, the latter being what we aim to quantify with the BKT. One participant who met the criteria for this study was unable to perform the BKT due to insufficient motor ability to reach across the test page, highlighting this circumstance. We suggest this limitation can be addressed by establishing minimum motor criteria for the BKT. Minimum motor criteria would establish that an individual possesses the motor ability to perform the reach, ideally leaving kinesthetic sense as the primary variable captured. The extent to which standardized table and chair heights affected BKT scores in this study is unknown. A limitation of this particular study is the small sample size; therefore, the results may not generalize to the population of individuals with mild and moderate stroke.

As a group, post-stroke participants in this study also performed significantly more poorly on the BKT with their *ipsilesional* UE than the comparison group suggesting that the BKT may be sensitive to subtle ipsilesional changes in kinesthetic awareness (Figure 1B). Ipsilesional impairment in sensorimotor performance^21^, manual dexterity^3^, ipsilesional reaching^22^, and grip force modulation^23^ have also been reported after unilateral stroke. While we can only speculate as to the sensorimotor processing problem that causes ipsilesional impairment, these data and other studies^5^^,^ 21 suggest that bilateral hemispheres likely contribute to normal kinesthetic performance. Additionally, these data call into question the concept of an 'unaffected' UE and highlight the importance of using healthy comparison groups for normative UE performance data.

The BKT has the potential to be a useful clinical tool with some additional development. Advantages of the BKT include that it is inexpensive, standardized, quantifiable, and quick to administer. The results from this study do not suggest there is a ceiling effect, unlike the Nottingham Sensory Assessment and the Fugl-Meyer Assessment^24^. Low RSEM values suggest BKT scores may provide a relatively precise estimate of the population. The simple instructions may limit the potential for confounding by cognitive impairment seen with other somatosensory measures. Future research should be directed at establishing validity, reliability, and the minimum clinically important difference in the post-stroke population. Minimum motor criteria should be established. Also of interest is whether BKT scores would be useful to predict motor recovery from stroke and whether the measure will be sensitive to change following UE rehabilitation.

The implications for improving identification of post-stroke kinesthetic impairment include enhanced understanding of the impairments that result in disordered reaching, improved assignment of rehabilitation treatments, and possible prediction of motor recovery. The data from this preliminary study suggest that, with further refinement, the BKT may emerge as a valuable clinical measure of kinesthetic awareness post-stroke.

## Clinical message

Inexpensive and quick to administer, the BKT may become a valuable clinical measure of post-stroke kinesthetic impairment.

## References

[1] World Health Organization - WHO (2008). The World Health Report 2008: Primary Health care: now more than
ever.

[2] Carey LM, Matyas TA (2011). Frequency of discriminative sensory loss in the hand after stroke in a
rehabilitation setting. J Rehabil Med.

[3] Nowak DA, Grefkes C, Dafotakis M, Küst J, Karbe H, Fink GR (2007). Dexterity is impaired at both hands following unilateral subcortical
middle cerebral artery stroke. Eur J Neurosci.

[4] Sommerfeld DK, von Arbin MH (2004). The impact of somatosensory function on activity performance and
length of hospital stay in geriatric patients with stroke. Clin Rehabil.

[5] Semrau JA, Herter TM, Scott SH, Dukelow SP (2013). Robotic identification of kinesthetic deficits after
stroke. Stroke.

[6] Nudo RJ (2007). Postinfarct cortical plasticity and behavioral
recovery. Stroke.

[7] United States (2014). Office of Management and Budget - OMB. General Services Administration - GSA.
The Brief Kinesthesia Test.

[8] Dunn W, Griffith JW, Morrison MT, Tanquary J, Sabata D, Victorson D (2013). Somatosensation assessment using the NIH Toolbox. Neurology.

[9] Taub E, Uswatte G, Mark VW, Morris DM, Barman J, Bowman MH (2013). Method for enhancing real-world use of a more affected arm in chronic
stroke transfer package of constraint-induced movement therapy. Stroke.

[10] Fullerton KJ, McSherry D, Stout RW (1986). Albert's test: a neglected test of perceptual neglect. Lancet.

[11] Williams PS, Basso DM, Case-Smith J, Nichols-Larsen DS (2006). Development of the hand active sensation test: reliability and
validity. Arch Phys Med Rehabil.

[12] Hunter JM, Mackin EJ, Callahan AD (1995). Rehabilitation of the hand: surgery and therapy.

[13] Bogard K, Wolf S, Zhang Q, Thompson P, Morris D, Nichols-Larsen D (2009). Can the Wolf Motor Function Test be streamlined?. Neurorehabil Neural Repair.

[14] Hodics TM, Nakatsuka K, Upreti B, Alex A, Smith PS, Pezzullo JC (2012). Wolf Motor Function Test for characterizing moderate to severe
hemiparesis in stroke patients. Arch Phys Med Rehabil.

[15] Uswatte G, Taub E, Morris D, Light K, Thompson PA (2006). The Motor Activity Log-28: assessing daily use of the hemiparetic arm
after stroke. Neurology.

[16] Mathiowetz V, Volland G, Kashman N, Weber K (1985). Adult norms for the Box and Block Test of manual
dexterity. Am J Occup Ther.

[17] Portney LG, Watkins MP (2007). Foundations of clinical research: applications to practice.

[18] Connell LA, Lincoln NB, Radford KA (2008). Somatosensory impairment after stroke: frequency of different deficits
and their recovery. Clin Rehabil.

[19] Borstad AL, Nichols-Larsen DS (2014). Assessing and treating Higher-level Somatosensory Impairments Post
Stroke. Top Stroke Rehabil.

[20] Goble DJ (2010). Proprioceptive acuity assessment via joint position matching: from
basic science to general practice. Phys Ther.

[21] Desrosiers J, Bourbonnais D, Bravo G, Roy P-M, Guay M (1996). Performance of the 'unaffected'upper extremity of elderly stroke
patients. Stroke.

[22] Pohl PS, Winstein CJ, Onla-Or S (1997). Sensory: motor control in the ipsilesional upper extremity after
stroke. NeuroRehabilitation.

[23] Quaney BM, Perera S, Maletsky R, Luchies CW, Nudo RJ (2005). Impaired grip force modulation in the ipsilesional hand after
unilateral middle cerebral artery stroke. Neurorehabil Neural Repair.

[24] Lima DH, Queiroz AP, Salvo GD, Yoneyama SM, Oberg TD, Lima NM (2010). Brazilian version of the Nottingham Sensory Assessment: validity,
agreement and reliability. Rev Bras Fisioter.

